# TOM40 regulates the progression of nasopharyngeal carcinoma through ROS-mediated AKT/mTOR and p53 signaling

**DOI:** 10.1007/s12672-023-00721-3

**Published:** 2023-06-23

**Authors:** Hong Ran, Jin Zhang, Xiaoxia Zeng, Zhen Wang, Peng Liu, Chenglin Kang, Shuqi Qiu, Xianhai Zeng, Peng Zhang

**Affiliations:** 1 Department of Graduate and Scientific Research, Zunyi Medical University Zhuhai Campus, Zhuhai, Guangdong China; 2 Department of Otorhinolaryngology, Longgang Otorhinolaryngology Hospital & Shenzhen Key Laboratory of Otorhinolaryngology, Shenzhen Institute of Otorhinolaryngology, No. 3004 Longgang Avenue, Shenzhen, Guangdong China; 3 Department of Otorhinolaryngology, The Second People’s Hospital of Yibin, Yibin, Sichuan China

**Keywords:** Nasopharyngeal carcinoma, TOM40, ROS, AKT, p53

## Abstract

Nasopharyngeal carcinoma (NPC) is a prevalent cancer in Southern China, North Africa, and Southeast Asia. The translocase of the outer membrane (TOM) 40 is a transporter of mitochondrial proteins, and is involved in ovarian cancer cell growth. However, its role in the progression of NPC is still unclear. We found that TOM40 levels were upregulated in NPC tissues and multiple NPC cell lines. In addition, high TOM40 expression in the tumor tissues was associated with poor overall survival and disease specific survival. TOM40 knockdown in the NPC cell lines inhibited their proliferation in vitro and in vivo. Furthermore, TOM40 silencing also increased intracellular production of reactive oxygen species (ROS) and decreased mitochondrial membrane potential (MMP). Mechanistically, the anti-tumor effects of TOM40 silencing were dependent on the inhibition of AKT/mTOR signaling and activation of p53 signaling. To summarize, TOM40 mediates NPC progression through ROS-mediated AKT/mTOR and p53 signaling. Our findings highlight the potential of TOM40 as a therapeutic target for NPC.

**Supplementary Information:**

The online version contains supplementary material available at 10.1007/s12672-023-00721-3.

## Introduction

Nasopharyngeal carcinoma (NPC) is a type of head and neck squamous cell carcinoma (HNSCC) that originates in the mucosal epithelium of the nasopharynx [1]. It is associated with significantly high regional incidence, ethnic susceptibility, and high familial incidence [2]. According to World Health Organization reports, 80% of the global nasopharyngeal cancer cases occur in China. Since the early stage of NPC is insidious, 97% of the NPC patients are clinically diagnosed in the middle and late stages of the disease [3]. Although NPC is sensitive to radio/chemotherapy, the 5-year overall survival rate is only 32%-62% [4, 5]. Therefore, it is crucial to understand the molecular basis of NPC progression in order to identify new therapeutic targets and develop more effective drugs.

Mitochondrial biogenesis and quality control are enhanced in cancer cells to sustain their rapid growth and proliferation [5]. The translocase of the outer membrane (TOM) transports proteins from the cytosol to the mitochondria, which is essential for the latter’s functions [6]. TOM forms a heteromeric channel comprising of TOM40, TOM70, TOM20, TOM22, TOM5, TOM6 and TOM7. TOM40 is an evolutionarily conserved 19 stranded β-barrel protein in eukaryotes [7], and has been implicated in neurodegenerative diseases such as Parkinson’s disease and Alzheimer’s disease [8, 9]. One study reported that TOM40 is overexpressed in ovarian cancer tissues, and promotes the growth of the malignant cells by modulating mitochondrial energy metabolism [10]. However, the correlation between TOM40 and NPC is still unknown, and the potential molecular mechanisms remain to be elucidated.

The reactive oxygen species (ROS) produced during mitochondrial oxidative phosphorylation acts as a two-edged sword in cancer progression depending on its concentration and distribution [11]. While abnormal ROS levels enhance cancer cell proliferation, excessive ROS accumulation would trigger cell death [11]. ROS mediates cell proliferation via the PI3K-AKT and MAPK-ERK signaling pathways. Deng et al. reported that dexamethasone, a type of glucocorticoid, increased intracellular ROS levels and inhibited the PI3K-AKT-GSK3β pathway in osteoblasts and MC3T3-E1 cells, eventually leading to apoptosis [12]. Su et al. demonstrated that vitamin C suppressed the proliferation of thyroid cancer cells and enhanced apoptosis through ROS-dependent inhibition of MAPK-ERK and PI3K-AKT pathways [13]. Another study showed that the ginsenoside Rh1 triggered ROS-induced apoptosis and cell cycle arrest by inhibiting the PI3K-AKT pathway [14]. Kaempferol suppressed pancreatic cancer cell growth through ROS-dependent apoptosis, which was involved in transglutaminase mediated AKT-mTOR pathway [15]. In addition, the increase in ROS production disrupts the redox balance in the cellular environment and promotes the over-expression and phosphorylation of p53, the upstream signaling molecule of apoptosis [16]. Wang et al. found that indoleamine 2,3-dioxygenase 1 (IDO1) enhances chemoresistance of ovarian cancer cells through inhibition of cisplatin-induced ROS accumulation and activation of p53 signaling pathway [17]. Therefore, TOM40 may affect the progression of NPC through ROS-related signaling pathways.

The aim of this study was to explore the role of TOM40 in the development of NPC. We detected high expression levels of TOM40 in NPC tissues and cell lines, and found that the upregulation of TOM40 in the tumors was associated with poor prognosis. Knocking down TOM40 expression in NPC cells inhibited their proliferation in vitro and in vivo through ROS-dependent AKT/mTOR and p53/p21 signaling pathways. Our findings provide new insights into the mechanisms driving NPC progression and identify TOM40 as a potential therapeutic target.

## Material and methods

### Cell culture

The human NPC cell lines CNE2, HONE1, 5-8F, 6-10B and C666-1, and the immortalized nasopharyngeal epithelium cell line NP69 have been described previously [18]. NPC cell lines were cultured in DMEM or RPMI 1640 medium supplemented with 10% FBS (Gibco). The NP69 cells were cultured in serum-free keratinocyte growth medium supplemented with 5 μg/L epidermal growth factor and 50 mg/L bovine pituitary extract (Gibco). All cells were cultured at 37 ℃ in a humidified incubator (Thermo Fisher Scientific) with 5% CO_2_.

### Immunohistochemistry

Immunohistochemical staining was performed as previously described [19]. Briefly, the slides were incubated overnight with anti-TOM40 antibody (Proteintech) at 4 ℃ in a humidified chamber, and processed with the GTVision III Detection System/Mo&Rb Kit (Gene Tech) to develop color.

### RNA isolation and real-time PCR

Total RNA was isolated using the RNeasy Mini Kit (Qiagen) and reverse transcribed using RT Master Mix for qPCR kit (MedChemExpress). Real-time PCR was performed using SYBR Green qPCR Master Mix (MedChemExpress) on the ABI7500 FAST Real Time PCR system (Applied Biosystems). ACTB was used as the internal control. The primer sequences were as follows: TOM40 forward 5′- CGAAGTTTGTGAACTGGCAGGTG -3′; reverse 5′- AAGGCGTGATGCTCTGGAGGTA-3′; MFN1 forward 5′- GGTGAATGAGCGGCTTTCCAAG-3′, reverse 5′- TCCTCCACCAAGAAATGCAGGC-3′; MFN2 forward 5′- ATTGCAGAGGCGGTTCGACTCA-3′, reverse 5′- TTCAGTCGGTCTTGCCGCTCTT-3′; DRP1 forward 5′-GATGCCATAGTTGAAGTGGTGAC-3′, reverse 5′- CCACAAGCATCAGCAAAGTCTGG-3′; ACTB forward 5′-CACCATTGGCAATGAGCGGTTC-3′, reverse 5′-AGGTCTTTGCGGATGTC CACGT-3′.

### siRNA transfection

The TOM40 siRNA sequences were provided by Shanghai GenePharma Co. Ltd. Cells were transfected with the respective siRNAs using GP-transfect-Mate (GenePharma) according to the manufacturer’s protocol. The siRNA sequences targeting TOM40 were as follows: siTOM40#1: 5′-GCAAGGAGCUGUUUCCCAUTT-3′, siTOM40#2: 5′-GGUUGGCAACGGUAACGUUTT-3′.

### Western blotting

The protein fraction was extracted from the cultured cells using RIPA lysis buffer (Beyotime Biotech). Equal amounts of protein per sample (20 μg) were separated by SDS-PAGE, and then transferred to a PVDF membrane (Millipore). After blocking with 5% BSA in TBST for 1 h at room temperature, the blots were incubated overnight with primary antibodies at 4 ℃ with constant shaking. Following incubation with HRP-conjugated secondary antibodies (Cell Signaling) at room temperature for 2 h, the positive bands were developed using the Pierce™ ECL Western Blotting Substrate (Thermo Scientific).

### Immunofluorescence staining

Cells were cultured in confocal dishes for the stipulated duration, fixed with 4% paraformaldehyde (Sigma-Aldrich) in PBS (Beyotime) for 15 min, and then blocked with 5% goat serum (Beyotime) in PBS for 30 min at room temperature. The cells were incubated overnight with anti-TOM40 antibody (1:200) (Proteintech) at 4 ℃, followed by incubation with Alexa Fluor-488 donkey anti-rabbit IgG (1:500) (Invitrogen) for 1 h at room temperature. After counterstaining with DAPI (Beyotime), the cells were imaged with a Leica TCS SP5 confocal microscope.

### Cell viability assay

To determine cell viability, the transfected cells were seeded in 96 well plates and cultured for varying durations. At pre-determined time points, 10 μL Cell Counting Kit-8 reagent (MedChemExpress) was added to each well, and the cells were incubated for 2 h at 37 ℃. Absorbance was measured at 450 nm on a multi-mode plate reader (Molecular Devices).

### Colony forming assay

The cells were seeded in 6 well plates at the density of 500 cells per well. After 2 weeks of culturing, the ensuing colonies were fixed with 4% paraformaldehyde and stained with crystal violet. Colonies with more than 50 cells were counted.

### EdU assay

The proliferative capacity of the cells was also evaluated by EdU incorporation using the BeyoClick^™^ EdU Cell Proliferation Kit with Alexa Fluor 488 (Beyotime Biotech) according to the manufacturer’s protocol. Briefly, the cells were incubated with EdU for 2 h at 37 ℃, fixed with 4% paraformaldehyde for 15 min, and permeabilized with 0.3% Triton X-100 for 15 min. The suitably treated cells were incubated with the Click Reaction Mixture for 30 min away from light, and then stained with Hoechst 33342 for 10 min. Images were taken with a Leica TCS SP5 confocal microscope.

### ROS staining

The intracellular generation of ROS was measured using CMH2DCFDA (Molecular Probes) as previously described [20]. Briefly, cells were seeded in confocal dishes and incubated with CMH2DCFDA for 15 min at 37 ℃, followed by Hoechst 33342 staining for 10 min. Images were taken with a Leica TCS SP5 confocal microscope.

### JC-1 staining

The changes in mitochondrial membrane potential (MMP) were measured using the JC-1 Mitochondrial Membrane Potential Assay Kit (Beyotime). Briefly, cells were seeded in confocal dishes and incubated with the JC-1 staining working buffer for 20 min at 37 ℃. Images were taken with a Leica TCS SP5 confocal microscope.

### In vivo* xenograft study*

Nude mice were injected subcutaneously with CNE2 or HONE1 cells transduced with lentiviruses expressing TOM40 shRNA or scrambled control into their flanks. The tumor size was measured using the formula: size (mm^3^) = width^2^ × length/2. The animals were euthanized, and the tumors were extracted and weighed. All experiments (maximal tumor size/burden were permitted and not exceeded) were approved by the Animal Experimental Ethics Committee of Shenzhen Institute of Otorhinolaryngology and Use of Laboratory Animals published by the US National Institute of Health.

### Statistical analysis

Statistical analysis was performed using GraphPad Prism software version 5.0. Data were expressed as mean ± standard error of the mean (SEM) of at least three independent experiments. Student’s t-test was used to compare two groups, and multiple groups were compared using one-way ANOVA. Cell viability and tumor volume were examined by two-way ANOVA. *P* value < 0.05 was considered statistically significant.

## Results

### TOM40 is upregulated in NPC

**Fig. 1:**
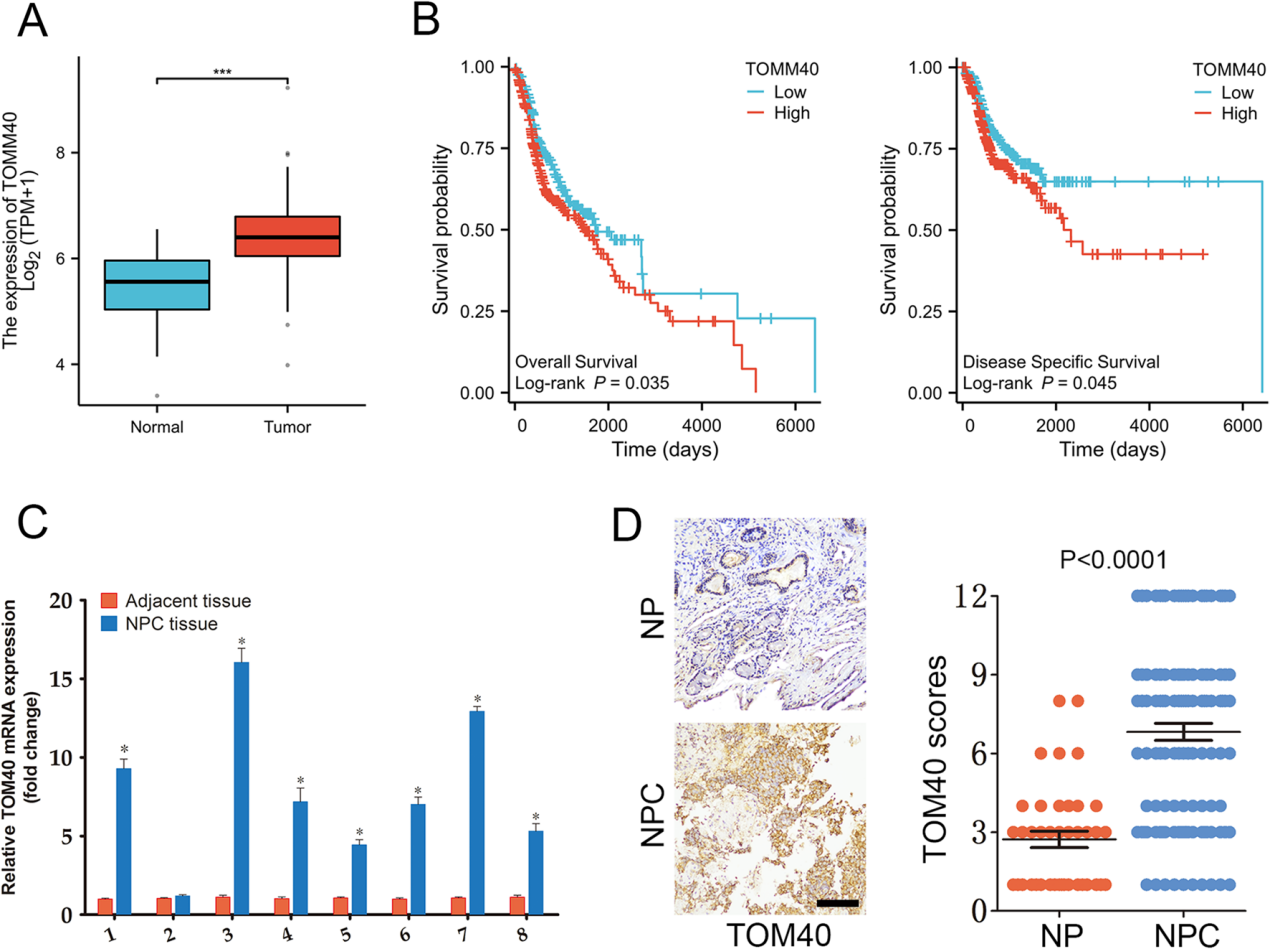
TOM40 expression in NPC tissues. **A** The relative TOM40 mRNA expression in the TCGA RNA-seq database. **B** Overall survival and disease specific survival of patients with high and low TOM40 expression. **C** Relative TRPV4 mRNA expression in 8 paired tumor and adjacent tissues from NPC patients. **D** Representative images and scores of TOM40 expression in NP (n = 40) and NPC tissues (n = 117). Scale bar = 50 μm. The data is the mean ± SEM of at least three independent experiments. * p < 0.05, versus NP or adjacent tissue

**Fig. 2:**
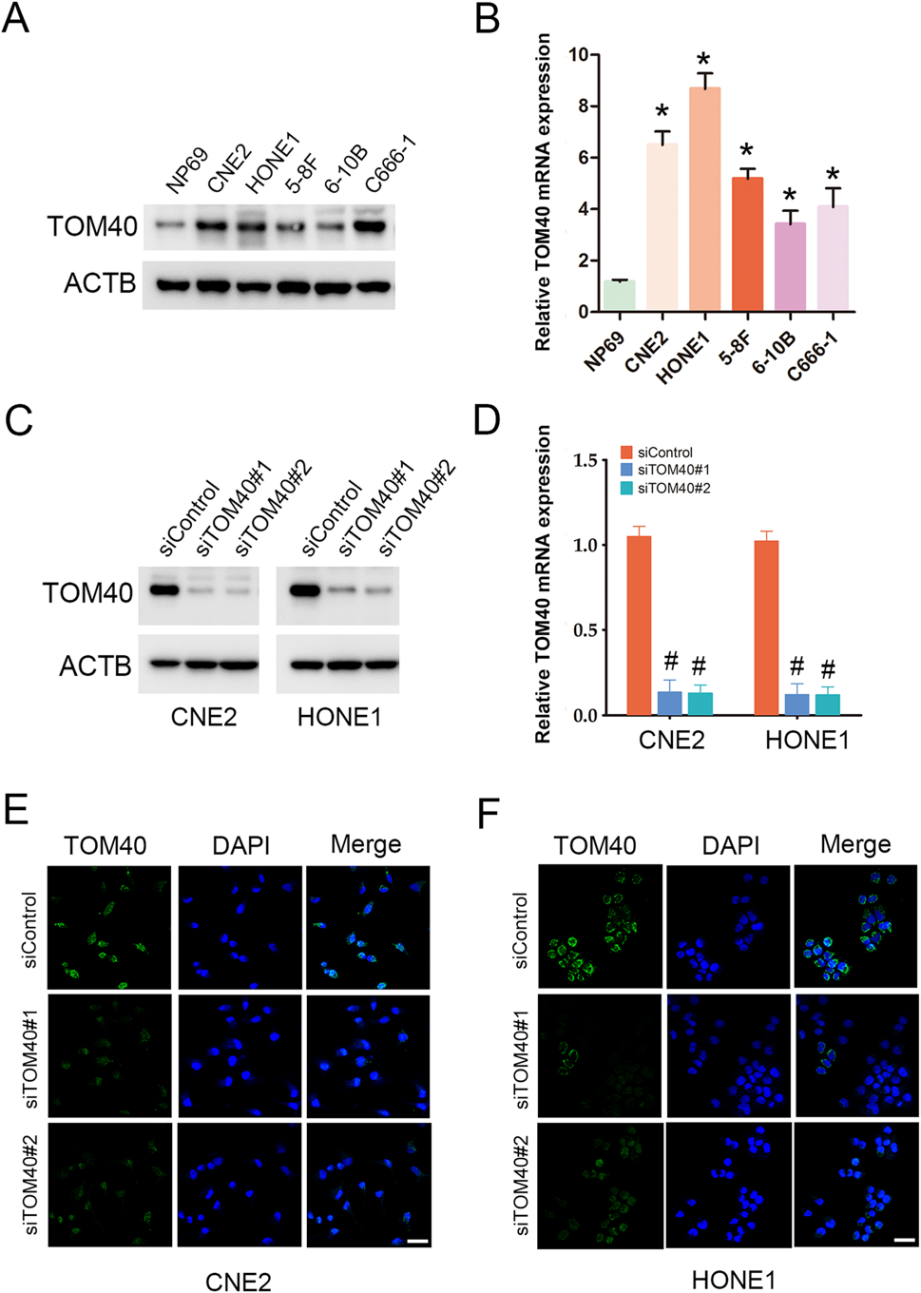
TOM40 expression in NPC cell lines. **A** and **B** TOM40 mRNA and protein expression in NP69, CNE2, HONE1, 5-8F, 6-10B and C666-1 cell lines. **C**–**F** TOM40 protein and mRNA expression in CNE2 and HONE1 cells transfected with siControl, siTOM40#1 or siTOM40#2. The data represent the mean ± SEM of at least three independent experiments. Scale bar = 20 μm * p < 0.05, versus NP69 or siControl

Analysis of the RNA-seq data from the Cancer Genome Atlas (TCGA) revealed that TOM40 mRNA was significantly upregulated in the HNSCC tissues compared to that in the normal tissues (Fig. 1A). Furthermore, patients with high TOM40 expression had a shorter overall survival (OS) and disease specific survival (DSS) compared to those with low TOM40 expression (Fig. 1B). To further confirm TCGA data, we examined TOM40 mRNA expression in matched tumor and adjacent normal tissue specimens obtained from 9 NPC patients by real-time PCR. As shown in Fig. 1C, TOM40 mRNA expression was higher in the NPC tissues compared to adjacent tissues (Fig. 1C). Furthermore, immunostaining of NPC (n = 117) and nasopharyngitis (NP) (n = 40) tissues indicated that TOM40 protein expression was higher in the NPC tissues compared to that in the NP tissues (Fig. 1D). Likewise, TOM40 mRNA and protein levels were significantly higher in multiple NPC cell lines (CNE2, HONE1, 5-8F, 6-10B and C666-1) compared to that in the nasopharyngeal epithelium cell line NP69 (Fig. 2A, B). Taken together, TOM40 is aberrantly upregulated in NPC tissues and cells.

### *TOM40 knockdown inhibited NPC cell growth *in vitro* and *in vivo

**Fig. 3:**
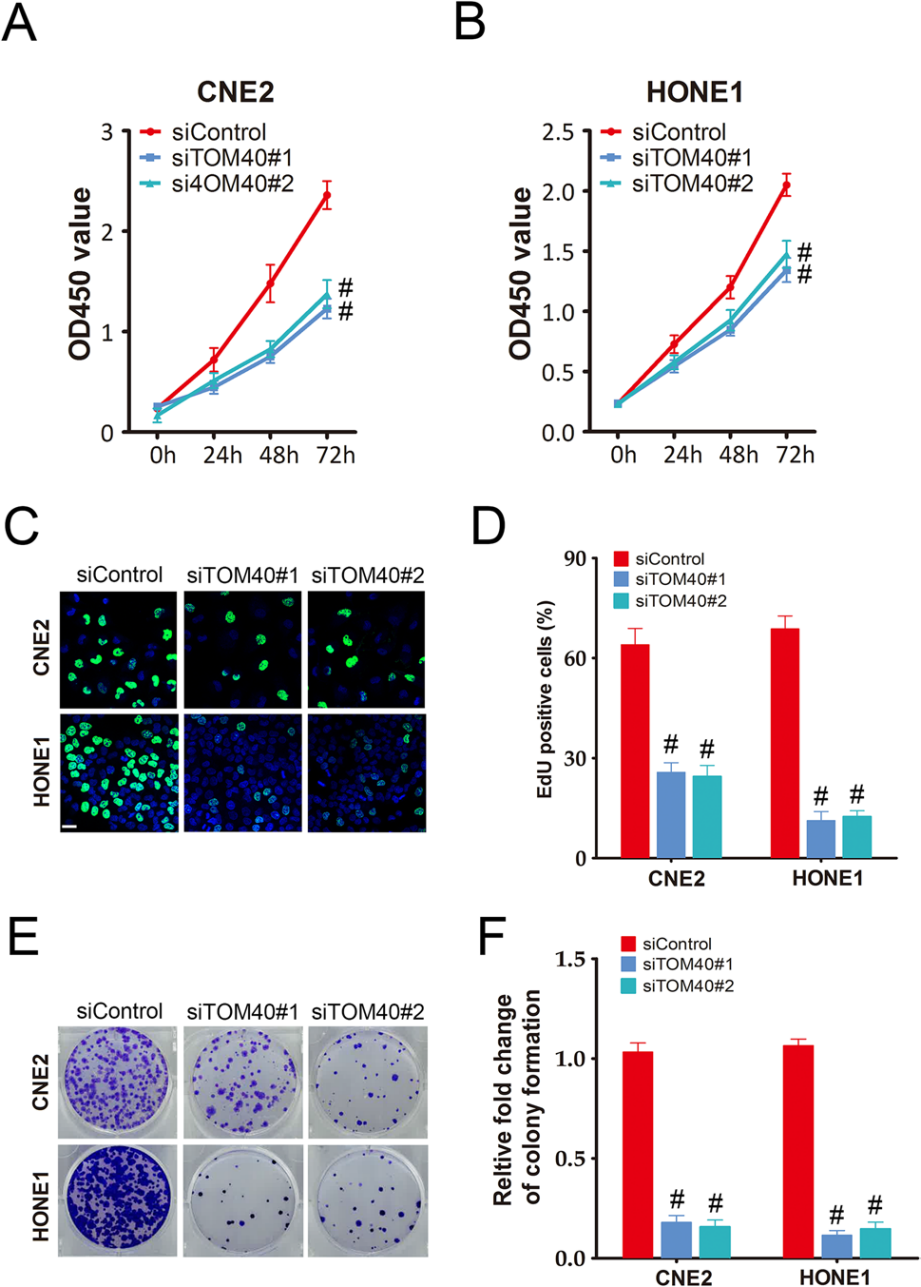
TOM40 knockdown inhibited proliferation of NPC cells in vitro*.* **A** and **B** Viability of CNE2 and HONE1 cells transfected with siControl, siTOM40#1 or siTOM40#2. **C** and **D** Representative images and number of EdU-positive proliferative CNE2 and HONE1 cells transfected with siControl, siTOM40#1 or siTOM40#2. Scale bar = 20 μm. **E** and **F** Representative images and number of colonies formed by CNE2 and HONE1 cells transfected with siControl, siTOM40#1 or siTOM40#2. The data represent the mean ± SEM of at least three independent experiments. * p < 0.05, versus siControl

**Fig. 4:**
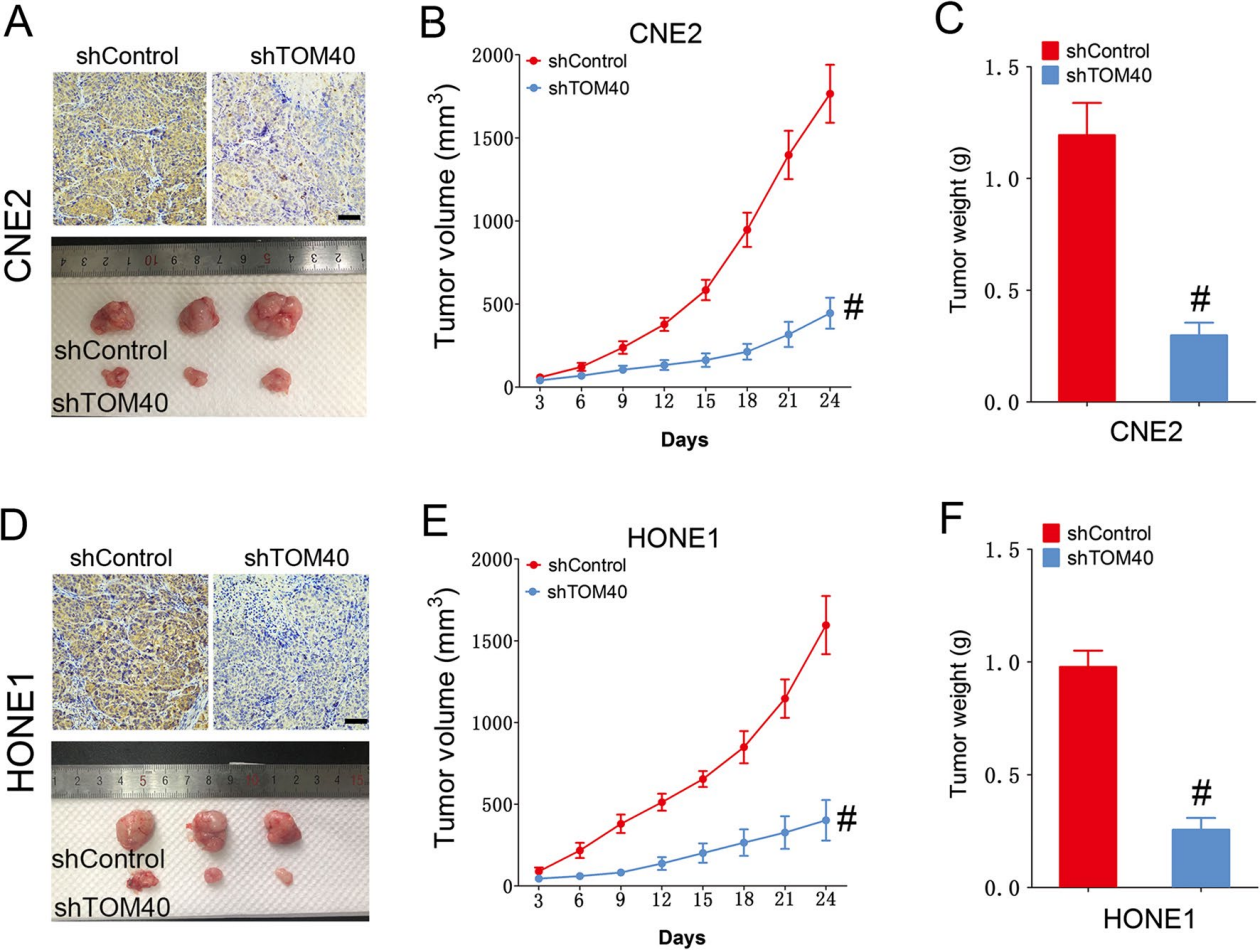
TOM40 knockdown inhibited proliferation of NPC cells in vivo*.* **A** Representative images of tumors and tissues immuno-stained for TMO40 from the xenografts of CNE2 cells transfected with shControl or shTOM40. **B** Volume of CNE2 xenografts from the indicated groups. **C** Weight of CNE2 xenografts from the indicated groups. **D** Representative images of tumors and tissues immuno-stained for TMO40 from the xenografts of HONE1 cells transfected with shControl or shTOM40. **E** Volume of HONE1 xenografts from the indicated groups. **F** Weight of HONE1 xenografts from the indicated groups. Scale bar = 50 μm. The data represent the mean ± SEM of at least three independent experiments. * p < 0.05, versus shControl

To further evaluate the role of TOM40 in the progression of NPC, we knocked down TOM40 in the CNE2 and HONE1 cells using two siRNA constructs (siTOM40#1 and siTOM40#2). Both siRNAs achieved significant suppression of TOM40 protein and mRNA in the NPC cell lines (Fig. 2C–F). Furthermore, TOM40 silencing significantly decreased the viability of the CNE2 and HONE1 cells as determined by the CCK-8 assay (Fig. 3A, B). The impact of TOM40 knockdown on the proliferation of NPC cells was evaluated by EdU incorporation and colony formation assays. The number of EdU-positive proliferative cells (green) was markedly reduced following transfection with siTOM40#1 and siTOM40#2 (Fig. 3C, D). Consistent with this, TOM40 knockdown also decreased the number of colonies formed by CNE2 and HONE1 cells compared to that by the respective controls (Fig. 3E, F). To validate the in vitro results, we established a xenograft model by subcutaneously transplanting NPC cells transfected with shTOM40 or shControl into nude mice. As shown in Fig. 4A and supplementary Fig. 1, TOM40 protein expression was decreased in xenograft tissues generated from the CNE2 cells with TOM40 knockdown. In addition, the tumor size and weight derived from TOM40-knockdown CNE2 cells were significantly lower compared to that in the control group (Fig. 4A–C). Likewise, inhibition of TOM40 expression in the HONE1 cells also attenuated the tumor size and weight (Fig. 4D–F). Taken together, TOM40 knockdown inhibited the growth of NPC cells in vitro and in vivo.

### TOM40 knockdown induced mitochondrial dysfunction in NPC cells

**Fig. 5:**
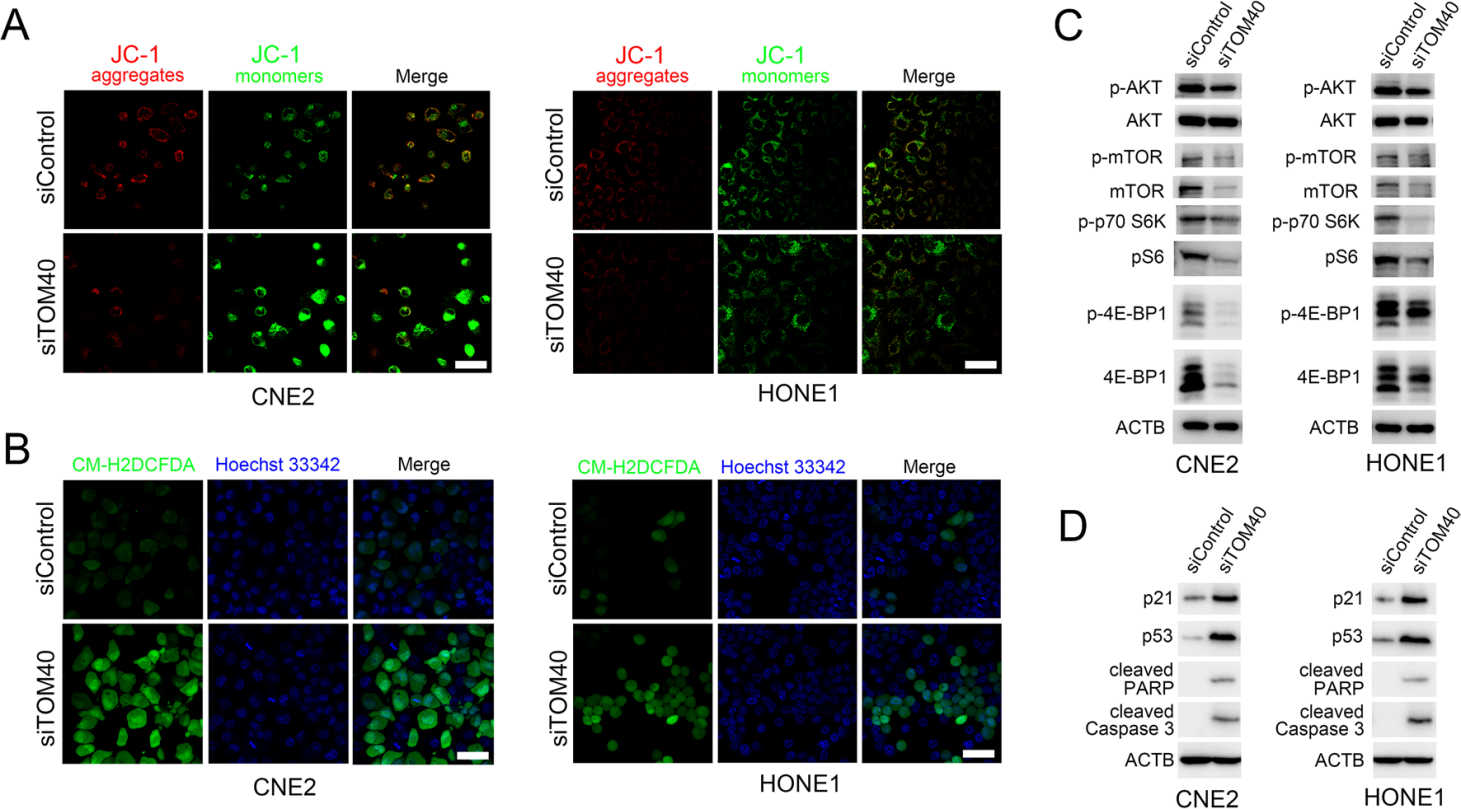
TOM40 knockdown induced mitochondrial dysfunction, AKT/mTOR signaling inhibition and p53 signaling activation. **A** The representative images of CNE2 and HONE1 cells stained with JC-1 showing changes in MMP following TOM40 knockdown. **B** Representative images of control and TOM40-knockdown CNE2 and HONE1 cells stained with the CMH2DCFDA probe showing ROS levels. **C** Immunoblot showing levels of p-AKT, AKT, p-mTOR, mTOR, p-p70S6K, pS6, p-4E-BP1, 4E-BP1and ACTB in the indicated groups. **D** Immunoblot showing levels of p21, p53, cleaved PARP, cleaved Caspase3 and ACTB in the indicated groups. Scale bar = 50 μm

To explore the effect of TOM40 knockdown on mitochondrial function, we measured the MMP in NPC cells transfected with shTOM40 or shControl using the JC-1 dye. When the MMP is high, JC-1 aggregates in the matrix and emits red fluorescence. In case of low membrane potential, JC-1 remains in the monomeric state and emits green fluorescence. The cells with TOM40 knockdown showed intense green fluorescence as opposed to the control cells, indicating low MMP (Fig. 5A). We also measured intracellular ROS generation using the CM-H2DCFDA fluorescent probe, and found that knocking down TOM40 led to a significant increase in ROS levels in the NPC cells (Fig. 5B). Moreover, mRNA expression of MFN1 and MFN2 was decreased and DRP1 mRNA level was increased in TOM40 knockdown NPC cells (Supplementary Fig. 2). Taken together, these results suggested that TOM40 knockdown may induce mitochondrial dysfunction in NPC cells by decreasing membrane potential and enhancing ROS production.

### *TOM40 knockdown inhibited NPC cell growth *via* inactivation of the ROS-dependent AKT/mTOR and p53 signaling pathways*

**Fig. 6:**
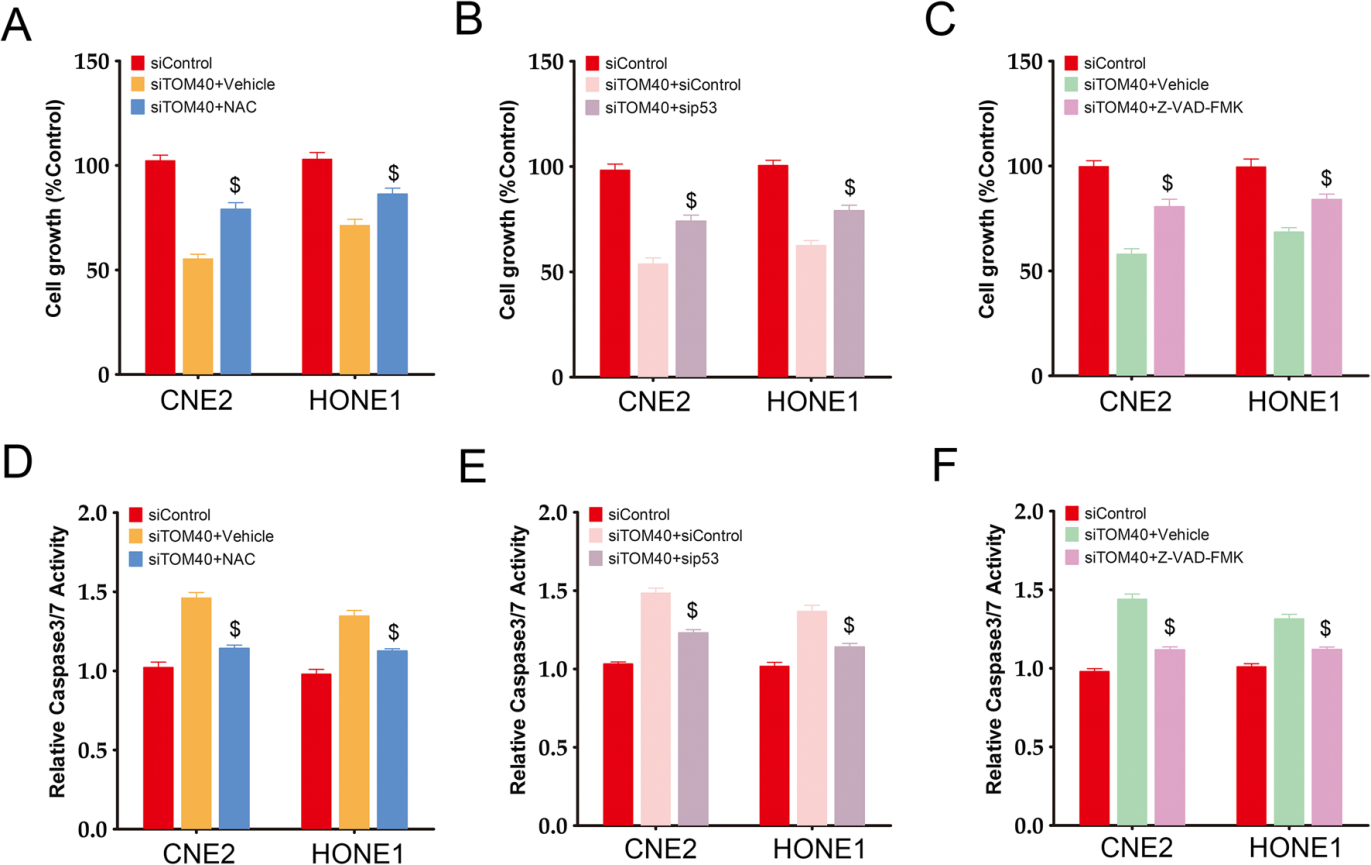
TOM40 knockdown inhibited cell growth through ROS-mediated AKT/mTOR and p53 signaling. **A**–**C** Viability of TOM40 knockdown CNE2 and HONE1 cells treated with NAC, sip53 and Z-VAD-FMK. **D–F** Caspase 3/7 activities in TOM40 knockdown CNE2 and HONE1 cells treated with NAC, sip53 and Z-VAD-FMK. The data represent the mean ± SEM of at least three independent experiments. $ p < 0.05, versus siTOM40 + Vehicle

Given that the AKT/mTOR signaling pathway plays an important role in NPC progression [21], we determined whether the effects of TOM40 knockdown in the NPC cells are mediated via this pathway. To this end, we evaluated the phosphorylation of AKT, mTOR, p70S6K and 4E-BP1 in CNE2 and HONE1 cells transfected with shTOM40 or shControl. As shown in Fig. 5C, TOM40 knockdown decreased the levels of phosphorylated AKT, mTOR, p70S6K, pS6 and 4E-BP1, suggesting inactivation of the AKT/mTOR signaling pathway. The p53/p21 pathway acts downstream of ROS signaling in NPC cells and is known to trigger apoptosis [16, 22]. We found that inhibition of TOM40 increased p21 and p53 levels, along with that of cleaved PARP and cleaved caspase 3, which lie downstream of p53 signaling. Furthermore, the ROS scavenger NAC significantly increased the viability of NPC cells with TOM40 knockdown (Fig. 6A). Likewise, knockdown of p53 and inhibition of pan-caspase activity with Z-VAD-FMK also reversed the effects of TOM40 knockdown and restored NPC cell viability (Fig. 6B, C). Moreover, we found that inhibition of ROS/p53/caspase signaling significantly suppressed caspase3/7 activity in the TOM40-knockdown NPC cells (Fig. 6D–F). These results suggest that TOM40 knockdown inhibits NPC cell growth through ROS-dependent AKT/mTOR and p53/p21 signaling pathways.

## Discussion

NPC has a unique geographical distribution, and is particularly prevalent in Southern China, North Africa, and Southeast Asia [1]. The primary approaches for the clinical management of NPC are radiotherapy and chemotherapy, which can damage normal tissues and reduce the quality of life of the patients. Therefore, it is urgent to discover novel therapeutic targets for effective treatment of NPC. In this study, we confirmed the high expression of TOM40 in NPC tissues and cell lines, and found that TOM40 silencing caused a significant reduction in the proliferative capacity of NPC cells in vivo and in vitro. Furthermore, TOM40 knockdown inhibited the growth of NPC cells by inactivating the ROS-dependent AKT/mTOR and p53/p21 signaling pathways. These findings shed light on the role of TOM40 in NPC progression, and highlight the therapeutic potential of targeting TOM40.

Functional mitochondria play an important role in cancer development [23]. TOM40 is a subunit of the TOM complex, which is responsible for importing proteins into the mitochondria and maintaining mitochondrial homeostasis [24]. TOM40-related mitochondrial dysfunction is involved in the pathogenesis of Parkinson’s disease, Alzheimer’s disease, and Huntington’s disease [24, 25]. Overexpressing TOM40 enhanced MMP and mitochondria-related bioenergetics, which prevented the cellular damage induced by β-amyloid [26]. One study showed that BAP31 interacts with TOM40 to from mitochondria-endoplasmic reticulum bridging complex in U2OS and HeLa cells, which is essential for mediating mitochondrial function [27]. Recently, Yang et al. demonstrated that TOM40 is upregulated in ovarian cancer tissues, and that higher TOM40 levels portended worse survival outcomes [10]. However, whether TOM40 plays a role in regulating progression of NPC is still unclear. In the present study, analysis of TCGA datasets revealed that TOM40 is overexpressed in HNSCC tissues and correlates with poor prognosis. In line with these results, TOM40 mRNA and protein were also overexpressed in human NPC tissues relative to paired adjacent normal tissues and nasopharyngitis tissues respectively. Likewise, high levels of TOM40 were also detected in multiple NPC cell lines compared to an immortalized nasopharyngeal epithelium cell line. TOM40 knockdown inhibited the proliferation of NPC cells in vitro, and impaired xenograft formation in vivo. These results suggested that TOM40 is involved in NPC progression.

Mitochondrial dysfunction drives excessive ROS production [11], which can inhibit cell proliferation and induce apoptosis in cancer cells [28]. Consistent with this, we found that TOM40 knockdown increased ROS production, whereas the antioxidant NAC reversed the inhibitory effect of TOM40 silencing on the proliferation of NPC cells. Given that TOM40 is a key subunit of the TOM complex in the mitochondrial membrane, we also examined the impact of TOM40 knockdown on the MMP in NPC cells. As expected, the MMP was lower in the cells with TOM40 knockdown compared to the controls, which suggested that the lack of TOM40 can trigger mitochondrial dysfunction. The AKT-mTOR signaling pathway is a key regulator of tumor cell growth, and can be inactivated by excessive ROS levels. Vitamin C-induced increase in ROS production in thyroid cancer cells promotes proteasomal degradation of AKT and inhibits cellular proliferation [13]. Celastrol suppressed glioma cell growth by increasing ROS production and dephosphorylating AKT and mTOR [29]. Likewise, itraconazole increased intracellular ROS levels and inhibited AKT/mTOR/S6K signaling in liver cancer cells, leading to growth inhibition [30]. Consistent with these findings, TOM40 knockdown in the NPC cell lines decreased the levels of phosphorylated AKT, mTOR, p70S6K and 4E-BP1, indicating that TOM40 may regulate NPC cell proliferation through the AKT-mTOR signaling pathway. In addition, mitochondrial ROS production activates the p53/p21 signaling pathway, which can decrease MMP and promote formation of the apoptotic complex [31, 32]. IDO1 overexpression in ovarian cancer cells suppressed the ROS-mediated p53-dependent cell death pathway, and protected the cells from cisplatin-induced apoptosis [17]. Furthermore, pulsed high-power microwave-induced ROS burst and p53 activation in the U87 MG cells decreased their viability and enhanced cell death [33]. Consistent with the fact that ROS/p53 is a cell death pathway, we found that TOM40 knockdown increased the expression of p53, p21, and activated caspase 3. Furthermore, we observed that p53 downregulation enhanced viability of NPC cells and repressed caspase activity in the setting of TOM40 knockdown. In line with this, inhibition of caspase activity also reversed the effects of TOM40 knockdown in NPC cells. Therefore, our results suggest that TOM40 regulates the progression of NPC through ROS-mediated AKT/mTOR and p53/p21 signaling.

In conclusion, TOM40 is overexpressed in NPC tissues and cell lines, and its inhibition repressed NPC cell growth through increased intracellular ROS accumulation. The anti-tumor effect of TOM40 knockdown was the result of impaired AKT/TOR signaling and activated p53/p21 signaling. Our findings suggest that TOM40 functions as an oncogene in NPC and is a potential therapeutic target.

## Supplementary Information

Additional file1

## Data Availability

The datasets generated during and/or analysed during the current study are available from the corresponding author on reasonable request.

## References

[1] Chen YP, Chan ATC, Le QT, Blanchard P, Sun Y, Ma J (2019). Nasopharyngeal carcinoma 2019. Lancet.

[2] Ding RB, Chen P, Rajendran BK, Lyu X, Wang H (2021). Molecular landscape and subtype-specific therapeutic response of nasopharyngeal carcinoma revealed by integrative pharmacogenomics 2021. Nat Commun.

[3] Lee HM, Okuda KS, Gonzalez FE, Patel V (2019). Current perspectives on nasopharyngeal carcinoma 2019. Adv Exp Med Biol.

[4] Sarmiento MP, Mejia MB (2014). Preliminary assessment of nasopharyngeal carcinoma incidence in the Philippines: a second look at published data from four centers 2014. Chin J Cancer.

[5] Zong WX, Rabinowitz JD, White E (2016). Mitochondria and cancer 2016. Mol Cell.

[6] Calvo SE, Clauser KR, Mootha VK (2016). MitoCarta2.0: an updated inventory of mammalian mitochondrial proteins 2016. Nucleic Acids Res.

[7] Taylor RD, McHale BJ, Nargang FE (2003). Characterization of *Neurospora* crassa Tom40-deficient mutants and effect of specific mutations on Tom40 assembly 2003. J Biol Chem.

[8] Soyal SM, Kwik M, Kalev O, Lenz S, Zara G (2020). A TOMM40/APOE allele encoding APOE-E3 predicts high likelihood of late-onset Alzheimer's disease in autopsy cases 2020. Mol Genet Genomic Med.

[9] Heinemeyer T, Stemmet M, Bardien S, Neethling A (2019). Underappreciated roles of the translocase of the outer and inner mitochondrial membrane protein complexes in human disease 2019. DNA Cell Biol.

[10] Yang W, Shin HY, Cho H, Chung JY, Lee EJ (2020). TOM40 Inhibits ovarian cancer cell growth by modulating mitochondrial function including intracellular ATP and ROS levels 2020. Cancers.

[11] Wang Y, Qi H, Liu Y, Duan C, Liu X (2021). The double-edged roles of ROS in cancer prevention and therapy 2021. Theranostics.

[12] Deng S, Dai G, Chen S, Nie Z, Zhou J (2019). Dexamethasone induces osteoblast apoptosis through ROS-PI3K/AKT/GSK3beta signaling pathway 2019. Biomed Pharmacother.

[13] Su X, Shen Z, Yang Q, Sui F, Pu J (2019). Vitamin C kills thyroid cancer cells through ROS-dependent inhibition of MAPK/ERK and PI3K/AKT pathways via distinct mechanisms 2019. Theranostics.

[14] Huynh DTN, Jin Y, Myung CS, Heo KS (2021). Ginsenoside Rh1 induces MCF-7 cell apoptosis and autophagic cell death through ROS-mediated Akt signaling 2021. Cancers.

[15] Wang F, Wang L, Qu C, Chen L, Geng Y (2021). Kaempferol induces ROS-dependent apoptosis in pancreatic cancer cells via TGM2-mediated Akt/mTOR signaling 2021. BMC Cancer.

[16] Liu B, Chen Y, St Clair DK (2008). ROS and p53: a versatile partnership 2008. Free Radic Biol Med.

[17] Wang H, Luo Y, Ran R, Li X, Ling H (2022). IDO1 Modulates the sensitivity of epithelial ovarian cancer cells to cisplatin through ROS/p53-dependent apoptosis 2022. Int J Mol Sci.

[18] Zhang P, Li K, Wang Z, Wu Y, Zhang H (2022). Transient receptor potential vanilloid type 4 (TRPV4) promotes tumorigenesis via NFAT4 activation in nasopharyngeal carcinoma 2022. Front Mol Biosci.

[19] Liu XY, Zheng CB, Wang T, Xu J, Zhang M (2020). SPZ1 promotes deregulation of Bim to boost apoptosis resistance in colorectal cancer,2020. Clin Sci (Lond).

[20] Gao MR, Zhang P, Han J, Tang CL, Zhu YF (2022). Small molecule compound M12 reduces vascular permeability in obese mice via blocking endothelial TRPV4-Nox2 interaction 2022. Acta Pharmacol Sin.

[21] Luo H, Yu YY, Chen HM, Wu W, Li Y, Lin H (2019). The combination of NVP-BEZ235 and rapamycin regulates nasopharyngeal carcinoma cell viability and apoptosis via the PI3K/AKT/mTOR pathway 2019. Exp Ther Med.

[22] Oyang L, Ouyang L, Yang L, Lin J, Xia L (2023). LPLUNC1 reduces glycolysis in nasopharyngeal carcinoma cells through the PHB1-p53/c-Myc axis 2023. Cancer Sci.

[23] Wallace DC (2012). Mitochondria and cancer 2012. Nat Rev Cancer.

[24] Pitt AS, Buchanan SK (2021). A Biochemical and structural understanding of TOM complex interactions and implications for human health and disease 2021. Cells.

[25] Gottschalk WK, Lutz MW, He YT, Saunders AM, Burns DK (2014). The Broad Impact of TOM40 on Neurodegenerative Diseases in Aging. J Parkinsons Dis Alzheimers Dis.

[26] Zeitlow K, Charlambous L, Ng I, Gagrani S, Mihovilovic M (2017). The biological foundation of the genetic association of TOMM40 with late-onset Alzheimer's disease 2017. Biochim Biophys Acta Mol Basis Dis.

[27] Namba T (2019). BAP31 regulates mitochondrial function via interaction with Tom40 within ER-mitochondria contact sites 2019. Sci Adv.

[28] Srinivas US, Tan BWQ, Vellayappan BA, Jeyasekharan AD (2019). ROS and the DNA damage response in cancer 2019. Redox Biol.

[29] Liu X, Zhao P, Wang X, Wang L, Zhu Y (2019). Celastrol mediates autophagy and apoptosis via the ROS/JNK and Akt/mTOR signaling pathways in glioma cells 2019. J Exp Clin Cancer Res.

[30] Wang W, Dong X, Liu Y, Ni B, Sai N (2020). Itraconazole exerts anti-liver cancer potential through the Wnt, PI3K/AKT/mTOR, and ROS pathways 2020. Biomed Pharmacother.

[31] Kumar D, Basu S, Parija L, Rout D, Manna S (2016). Curcumin and ellagic acid synergistically induce ROS generation, DNA damage, p53 accumulation and apoptosis in HeLa cervical carcinoma cells 2016. Biomed Pharmacother.

[32] Fridman JS, Lowe SW (2003). Control of apoptosis by p53 2003. Oncogene.

[33] Rana JN, Mumtaz S, Choi EH, Han I (2023). ROS production in response to high-power microwave pulses induces p53 activation and DNA damage in brain cells: Radiosensitivity and biological dosimetry evaluation 2023. Front Cell Dev Biol.

