# Supplementary material for: TOM40 regulates the progression of nasopharyngeal carcinoma through ROS-mediated AKT/mTOR and p53 signaling
Source: Discov Oncol. 2023 Jun 23;14:109. doi: 10.1007/s12672-023-00721-3 (PMC10290019; doi:10.1007/s12672-023-00721-3)
Supplement: Supplementary file 1 — Additional file1 [file 12672_2023_721_MOESM1_ESM.doc]

**Supplementary Material**


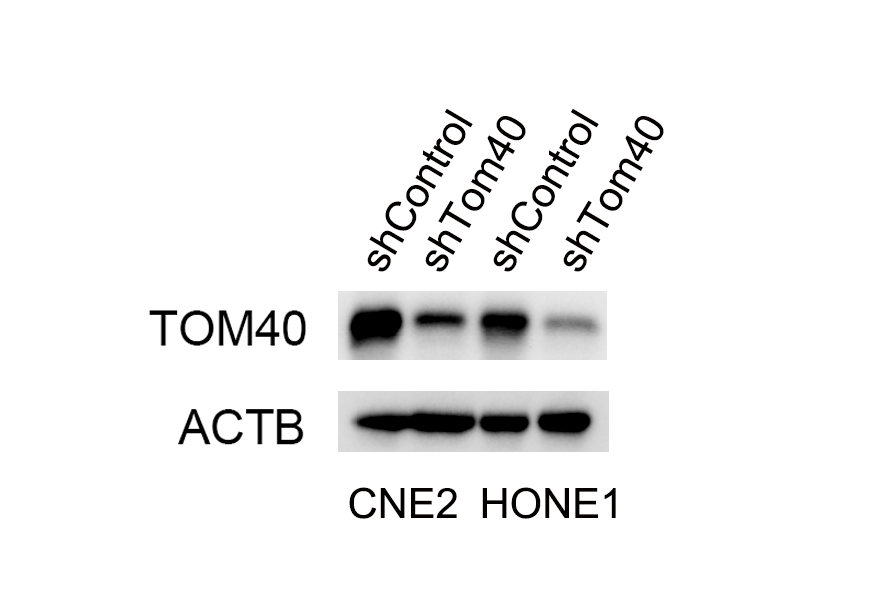


Supplementary Figure 1. The representative images of TOM40 protein expression in CNE2 and HONE1 cells transfected with shControl or shTOM40.


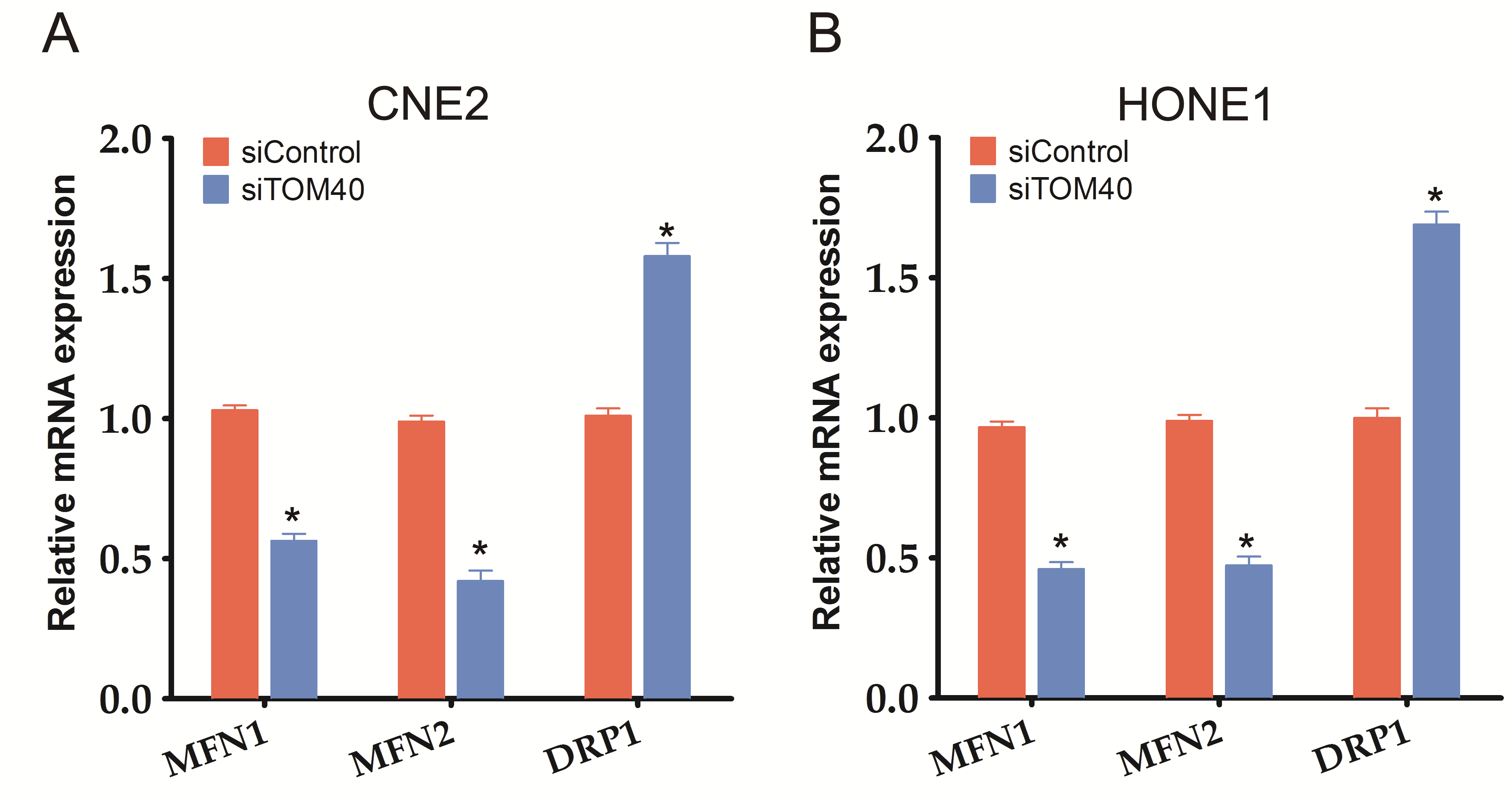


Supplementary Figure 2. (A and B) MFN1, MFN2 and DRP1 mRNA expression in CNE2 and HONE1 cells transfected with siControl or siTOM40. The data represent the mean ± SEM of at least three independent experiments. * p<0.05, versus siControl.
